# Stroke risk in arthritis: A systematic review and meta-analysis of cohort studies

**DOI:** 10.1371/journal.pone.0248564

**Published:** 2021-03-16

**Authors:** Wei Liu, Wei Ma, Hua Liu, Chunyan Li, Yangwei Zhang, Jie Liu, Yu Liang, Sijia Zhang, Zhen Wu, Chenghao Zang, Jianhui Guo, Liyan Li

**Affiliations:** 1 Institute of Neuroscience, Kunming Medical University, Kunming, Yunnan, China; 2 Department of Neurology, The Third People’s Hospital of Chengdu & The Affiliated Hospital of Southwest Jiaotong University, Chengdu, Sichuan, China; 3 Department of Neurology, Nanchong Central Hospital & The Second Clinical Medical College, North Sichuan Medical College, Nanchong, Sichuan, China; 4 Second Department of General Surgery, First People’s Hospital of Yunnan Province, Kunming, Yunnan, China; German Centre for Neurodegenerative Diseases Site Munich: Deutsches Zentrum fur Neurodegenerative Erkrankungen Standort Munchen, GERMANY

## Abstract

**Background and objective:**

Stroke is a major contributor to the global burden of disease. Although numerous modifiable risk factors (RF) for stroke have been identified, some remain unexplained. Increasing studies have investigated stroke risk in arthritis, but their results are inconsistent. We aimed to synthesize, quantify, and compare the risk of stroke for the major types of arthritis in cohort studies by using a systematic review and meta-analysis approach.

**Methods:**

We searched Chinese and English databases to identify relevant studies from inception to April 30, 2020. Only studies adjusting at least for age and sex were included. We calculated pooled effect estimates for relative risk (RR) and 95% confidence interval (CI) and identified potential sources of heterogeneity and publication bias.

**Results:**

A total of 1,348 articles were retrieved, and after an preliminary screening of titles and abstracts, 69 were reviewed for full text, and finally, 32 met the criteria for meta-analysis. Stroke risk in arthritis was significantly increased in studies adjusting for age and sex (RR = 1.36, 95% CI: 1.27–1.46) and for at least one traditional risk factor (RR = 1.40, 95% CI: 1.28–1.54). The results of studies stratified by stroke subtype were consistent with the main finding (ischemic stroke: RR = 1.53, 95% CI: 1.32–1.78; hemorrhagic stroke: RR = 1.45, 95% CI: 1.15–1.84). In subgroup analysis by arthritis type, stroke risk was significantly increased in rheumatoid arthritis (RR = 1.38, 95% CI: 1.29–1.48), ankylosing spondylitis (RR = 1.49, 95% CI: 1.25–1.77), psoriatic arthritis (RR = 1.33, 95% CI: 1.22–1.45), and gout (RR = 1.40, 95% CI: 1.13–1.73) but not osteoarthritis (RR = 1.03, 95% CI: 0.91–1.16). Age and sex subgroup analyses indicated that stroke risk was similar by sex (women: RR = 1.47, 95% CI: 1.31–1.66; men: RR = 1.44, 95% CI: 1.28–1.61); risk was higher with younger age (<45 years) (RR = 1.46, 95% CI: 1.17–1.82) than older age (≥65 years) (RR = 1.17, 95% CI: 1.08–1.26).

**Conclusions:**

Stroke risk was increased in multiple arthritis and similar between ischemic and hemorrhagic stroke. Young patients with arthritis had the highest risk.

## Introduction

Clinically, stroke is a medical condition based on a relatively sudden loss of focal neurological function, broadly categorized as ischemic stroke (IS) or hemorrhagic stroke (HS) [1]. Stroke has become a major public health concern. According to the Global Burden of Disease Study 2016, stroke accounts for almost 5% of all disability-adjusted life-years and 10% of all deaths worldwide, engendering substantial physical and emotional consequences for patients and their families [2]. Thus, there is an urgent need to clearly understand stroke risk. A number of modifiable risk factors have been associated with most of the population attributable risk in stroke worldwide; these include hypertension, diabetes mellitus, obesity, hyperlipidemia, smoking, alcoholism, physical inactivity, diet, psychosocial factors, and cardiac causes [3]. However, studies on these traditional risk factors (RF) cannot fully explain the continuous increase in stroke risk.

There is growing evidence to suggest that inflammation plays a key role in many chronic diseases, including cardiovascular disease, cancer, chronic kidney disease, diabetes, and stroke [4–6]. Arthritis is a chronic inflammatory disease characterized by inflammation of the synovial tissue of the joint [7, 8]. It’s inflammation begins with the infiltration of inflammatory cells into the joint [9, 10]. These cells release enzymes, pro-inflammatory factors, and other cytokines that degrade and destroy synovial tissue [9–11], eventually leading to joint pain, deformity, and mobility limitation [12, 13]. The most common types of arthritis are rheumatoid arthritis (RA), osteoarthritis (OA), psoriatic arthritis (PsA), ankylosing spondylitis (AS), gout, and pseudogout [14].

Inflammatory mediators such as cytokines may be the critical factor linking arthritis and stroke. Arthritis involves the increased generation of inflammatory cytokines produced in the joints. These eventually spill into the circulation, where they can cause increased production of adhesion molecules and other proinflammatory molecules. This leads to monocyte and leukocyte adhesion to the endothelial cells of the vessel wall, followed by chemotaxis of these into vessel walls, which leads to atherosclerosis, and ultimately to vascular events such as stroke [15, 16].

Over the past few decades, many epidemiological studies have evaluated the association between arthritis and stroke risk; however, the results have been inconsistent. Wiseman et al. [17] performed a meta-analysis of epidemiological studies on stroke risk in various rheumatic diseases, including RA, AS, and gout. The results showed that the stroke risk of these diseases increased. However, the meta-analysis only included studies up to December 14, 2014. Since then, increasingly more epidemiological studies investigating the stroke risk in numerous types of arthritis have been conducted. However, the consistency and quality of these studies have not been well reviewed, which limits the objective understanding of stroke risk in arthritis.

In epidemiology, cohort studies are preferable to case-control studies and cross-sectional studies for investigating etiological relationships. Generally speaking, age and sex are common potential confounders that might affect the actual result. Adjusting them can focus more on the effect of exposure itself on the outcome. Therefore, we conducted a comprehensive meta-analysis of cohort studies adjusting at least for age and sex to assess stroke risk in multiple types of arthritis.

## Materials and methods

### Study design

Our review was conducted according to the PRISMA (Preferred Reporting Items for Systematic Reviews and Meta-Analysis) statement [18] and MOOSE (Meta-Analyses and Systematic Reviews of Observational Studies) guidelines [19]. Arthritis was used as an exposure and stroke as an outcome. The present research did not require the approval of an ethics committee and was not registered in any database.

### Data sources and searches

We searched MEDLINE, EMBASE, Cochrane Library, China National Knowledge Infrastructure (CNKI), Weipu, Wanfang, and SINOMed databases using MeSH terms and their entry terms from the initial to April 30, 2020. The example of MeSH terms search strategy in MEDLINE is as follows: ("Stroke"[Mesh]) AND ("Arthritis"[Mesh] OR "Arthritis, Rheumatoid"[Mesh] OR "Arthritis, Psoriatic"[Mesh] OR "Spondylitis, Ankylosing"[Mesh] OR "Gout"[Mesh] OR "Osteoarthritis"[Mesh] OR "Chondrocalcinosis"[Mesh]) AND ("Cohort Studies"[Mesh]). The complete search strategy is presented in S1 Appendix.

All articles searched were stored and managed using Endnote X9 software throughout the review process. We first pooled results from different databases, performed the duplication removal step, and then conducted title and abstract screening followed by full-text screening. In addition, we manually searched the reference lists of identified studies and relevant review articles to identify any studies that were missed in our preliminary search. Articles were not excluded on the basis of language criteria.

### Study selection

Studies that met the following criteria were considered: (i) cohort studies adjusting at least for age and sex; (ii) exposure factors of interest were the six major arthritis types, with clear diagnostic criteria: RA, OA, PsA, AS, gout, and pseudogout; (iii) the outcome of interest was stroke; and (iv) relative risk (RR) and its corresponding 95% CI were reported or the data made available for calculation. Studies that met the following criteria were excluded: (i) not a cohort study, such as a cross-sectional or case-control study; and (ii) the reported RR and 95% CI was not adjusted for age and sex.

If two or more articles were published for the same cohort study, we only included the article with the most detailed information or the longest follow-up period.

### Data extraction

Two investigators used a standard format to extract the following information independently from each study: first author, publication date, country, arthritis type, study design, data source, sample size, study period, age range, proportion of women participants, exposure and outcome validation, stroke subtype, effect estimate type, RR and 95% CI, and additional covariates.

### Quality assessment

We checked the quality of the included studies using the Newcastle-Ottawa Scale (NOS) [20]. NOS is a tool for evaluating the quality of observational studies in systematic reviews and meta-analyses. In the NOS, each study is categorized according to three methodological characteristics: selection of the study group (scale 0–4); comparability of the study group (scale 0–2); and determination of the exposure or results in case-control or cohort studies (scale 0–3). The highest score for each study is 9. Scores of 0–3, 4–6, and 7–9 are generally regarded as indicative of low, moderate, and high quality, respectively.

### Data synthesis and analysis

RR was used in the statistical analysis of cohort study to measure the strength of the association between the exposure and outcome. Owing to the low absolute risk of stroke, the four correlation indicators were expected to produce similar RR estimates: [standardized mortality rate (SMR), standardized incidence ratio (SIR), incidence rate ratio (IRR), and hazard ratio (HR)] [21]. Therefore, we used these together to assess stroke risk in arthritis, to ensure the comprehensiveness of the evaluation and to maximize statistical power. We used the DerSimonian and Laird random-effects model [22] to calculate the pooled RR and 95% CI for all types of arthritis, first in the studies adjusting only for age and sex, and then in studies adjusting for at least one of the following traditional RF: hypertension, diabetes, smoking, alcoholism, obesity, physical inactivity, and hyperlipidemia. Heterogeneity among studies was assessed using Cochrane’s Q statistic (significance level at *p* < 0.10) and the I^2^ statistic, reflecting the percentage of heterogeneity in the total effect variation. I^2^ < 25% was considered to indicate no heterogeneity and 25%-50% to indicate low, 50%-75% moderate, and > 75% high heterogeneity [23]. We conducted subgroup analyses to explore the source and magnitude of heterogeneity and examine the effect of different subgroups from a professional perspective. We also performed sensitivity analyses to test the robustness of the results by excluding each study and retesting changes in the size of the combined effect. The publication bias was evaluated using the Begg test and Egger test; *p* < 0.10 was considered statistically significant [24].

All analyses were conducted independently by two authors (WL and WM) using Stata V.14 (StataCorp LLC, College Station, TX, USA). Disagreements were resolved in discussion with a third researcher (HL) until consensus was reached.

## Results

### Literature search and study election

**Fig 1:**
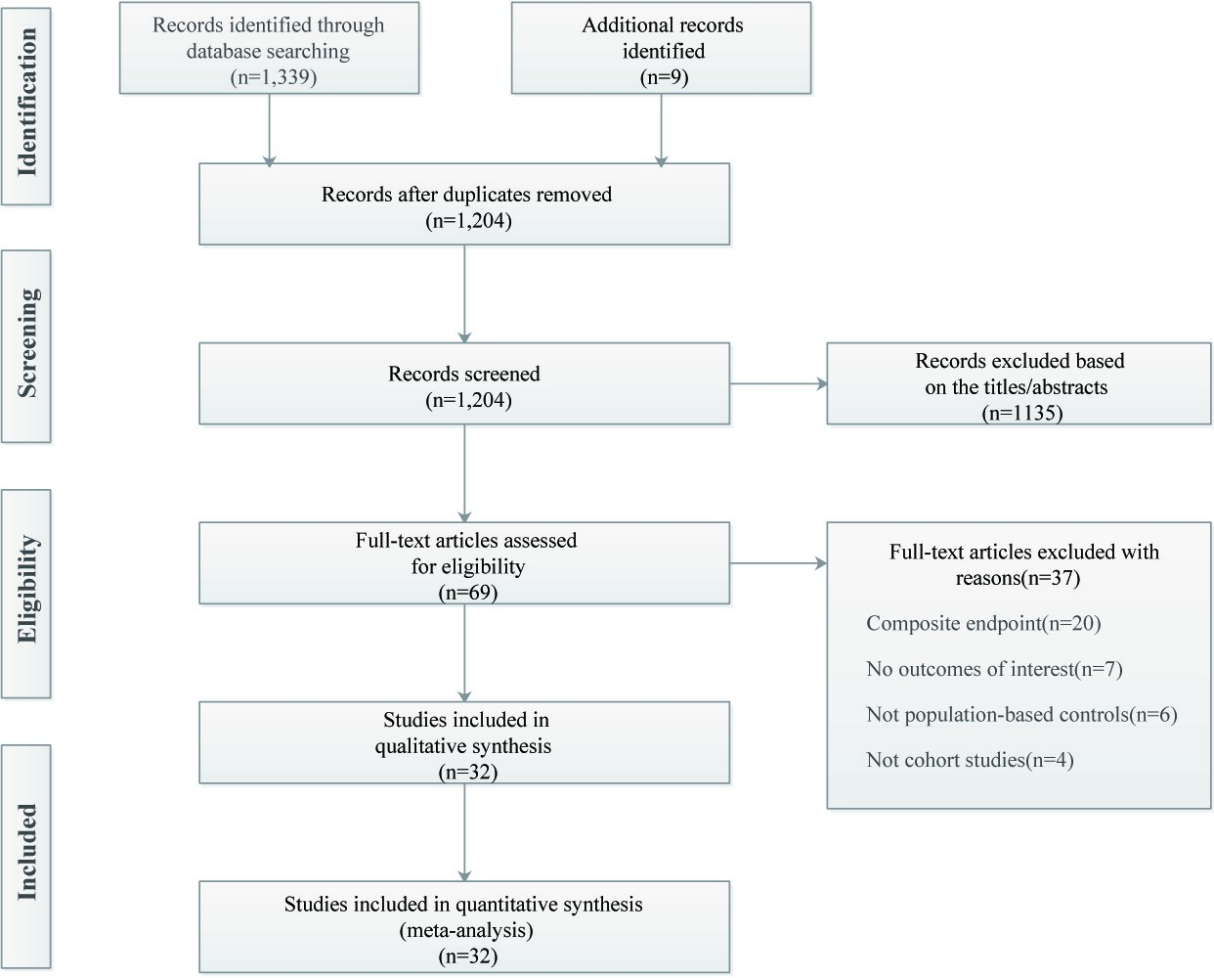
Flowchart of study selection for the meta-analysis (according to PRISMA statement).

The literature search and screening process are depicted in Fig 1. Our initial search returned 1,339 articles, and an additional 9 articles [25–33] were identified in a manual search. After excluding 144 duplicates, the title and abstract of 1,204 articles were reviewed, of which 1,135 were excluded. After a full-text review of the remaining 69 articles, 37 were omitted for various reasons; a total of 32 articles were finally included [25–56]. The completed PRISMA checklist is given in S1 Checklist.

### Study characteristics and quality

We summarized the main characteristics of the 32 included articles in Table 1. These articles were published from 1994 to 2019, over a span of 25 years. A total of 9 articles were conducted in North America [27, 32, 35, 36, 38, 39, 44, 49, 54], 14 in Europe [28–31, 33, 37, 40–43, 45, 50–52], and 9 in Asia [34, 46–48, 53, 55–58]. There were 10 prospective cohorts (PC) [27, 31, 34, 36, 39, 40, 42, 47, 49, 51] and 22 retrospective cohorts (RC) [28–30, 32, 33, 35, 37, 38, 41, 43–46, 48, 50, 52–58]. Because none of the articles on stroke risk in pseudogout met our inclusion criteria, we eventually only included five types of arthritis (OA, RA, PsA, AS, and gout) in the meta-analysis.

**Table 1:** Characteristics of included studies.

Study	First author,	Disease	Country	Design	Data	Study	Age	Women	Arthritis	Stroke	Exposure	Effect	NOS
NO.	Year(ref.no)				source	period	range	%	validation	validation	total	estimates	quality
1	Wolfe 1994 [25]	RA	USA&Canada	PC	ARAMIS	1965–1990	53(mean)	74.00%	ARA criteria	DC(ID-9)	3501	SMR	9(4/2/3)
2	Bjornadal 2002 [26]	RA	Sweden	RC	SHDR	1964–1994	NA	71.11%	ICD-7,8,9	ICD-7,8,9(DC)	46917	SMR	8(3/2/3)
3	Solomon 2003 [34]	RA	USA	PC	NHS	1978–1996	30–55	100.00%	1987 ACR	Medical records	NA	IRR	8(3/2/3)
4	Watson 2003 [27]	RA	UK	RC	GPRD	1987–2001	≥40	69.83%	Medical records	Medical records	11633	IRR	7(3/2/2)
5	Turesson 2004 [35]	RA	Sweden	RC	RA clinics	1997–1999	≥15	73.97%	1987ACR	ICD-9,10	1022	SMR	7(4/1/2)
6	Solomon 2006 [36]	RA	Canada	RC	BCLHD	1999–2003	≥18	71.13%	ICD-9	ICD-9	25385	IRR	7(3/2/2)
7	Bergstrom 2009 [28]	RA	Sweden	RC	RA clinic	1978–1985	≥16	79.10%	1958 ARA	ICD-9,10	148	SMR	7(3/2/2)
			Sweden	RC	RA clinic	1995–2002	≥16	77.60%	1987 ARA	ICD-9,10	161	SMR	7(3/2/2)
8	Semb 2010 [29]	RA	Sweden	PC	AMORIS	1985–1996	NA	68.92%	ICD-8,9,10	ICD-8,9,10	1779	IRR	8(4/1/3)
9	Szabo 2011 [30]	AS	Canada	RC	RAMQ	1996–2006	>19	43.87%	ICD-9	ICD-9	8616	SIR	7(3/1/3)
10	Brophy 2012 [31]	AS	UK	RC	HER	1999–2010	≥20	24.10%	EHR READ	EHR READ	1686	IRR	7(3/1/3)
11	Li 2012 [37]	PsA	USA	PC	NHS II	1991–2009	25–60	NA	Self-report	Medical record	NA	IRR	8(3/2/3)
12	Lindhardsen 2012 [38]	RA	Denmark	PC	DCVS	1997–2009	≥15	69.70%	ICD-10	ICD-10	18247	IRR	9(4/2/3)
13	Teng 2012 [32]	Gout	Singapore	PC	SCHS	1993–2009	49–62	34.30%	ICD-9	ICD-9	2117	HR	9(4/2/3)
14	Zoller 2012 [39]	AS	Sweden	RC	NSDR	1987–2008	NA	30.51%	ICD-7,8,9,10	ICD-9,10	3477	SIR	7(3/1/3)
		RA	Sweden	RC	NSDR	1987–2008	NA	72.92%	ICD-7,8,9,10	ICD-9,10	44611	SIR	7(3/1/3)
15	Holmqvist 2013 [40]	RA	Sweden	PC	NPR&SPR	1997–2009	≥16	NA	ICD-10	ICD-10	39065	HR	9(4/2/3)
16	Norton 2013 [41]	RA	UK	RC	ERAS	1986–1998	55.34(mean)	66.40%	ICD-10	ICD-10	1460	SIR	8(3/2/3)
17	Rahman 2013 [42]	OA	Canada	RC	BCLHD	1991–2009	≥20	60.00%	ICD-9,10	ICD-9,10	12745	IRR	7(3/2/2)
18	Seminog 2013 [43]	Gout	UK	RC	NHS	1999–2011	≥20	26.00%	ICD-7,8,9,10	ICD-10	202033	IRR	7(3/2/2)
			UK	RC	ORLS	1963–1998	≥20	27.00%	ICD-7,8,9,10	ICD-10	3174	IRR	8(3/2/3)
19	Keller 2014 [44]	AS	China	RC	LHID2000	2001	42.07(mean)	37.80%	ICD-9	ICD-9	2895	IRR	7(3/2/2)
20	Lin 2014 [45]	AS	China	PC	NHI	2001	18–45	26.20%	ICD-9	ICD-9	4562	HR	8(4/2/2)
21	Liou 2014 [46]	RA	China	RC	LHID2005	2004–2007	NA	71.30%	ICD-9	ICD-9	6114	HR	7(3/2/2)
22	Haugen 2015 [47]	OA	USA	PC	FHS	1990–2015	50–75	53.80%	Medical records	Medical records	186	IRR	8(3/2/3)
23	Ogdie 2015 [48]	RA	UK	RC	THIN	1994–2010	18–89	70.51%	EHR READ	EHR READ	41752	IRR	9(4/2/3)
		PsA	UK	RC	THIN	1994–2010	18–89	48.80%	EHR READ	EHR READ	8706	IRR	9(4/2/3)
24	Bengtsson 2017 [49]	AS	Sweden	PC	SNPR	2001–2009	18–99	31.90%	ICD-10	ICD-10	6448	IRR	8(4/1/3)
		PsA	Sweden	PC	SNPR	2001–2009	18–99	55.10%	ICD-10	ICD-10	16063	IRR	8(4/1/3)
25	Eriksson 2017 [50]	AS	Sweden	RC	NPR&CDR	2006–2011	≥18	32.00%	ICD-10	ICD-10	5248	IRR	7(3/2/2)
		RA	Sweden	RC	NPR&CDR	2006–2011	63.3(mean)	73.00%	ICD-10	ICD-10	35499	IRR	7(3/2/2
26	Hsu 2017 [51]	OA	China	RC	LHID2000	2002–2003	20–90	59.60%	ICD-9	ICD-9	43635	IRR	7(3/1/3)
27	So 2017 [52]	AS	Canada	RC	BC clinic	1990–2012	>18	48.70%	ICD-9,10	ICD-9,10	7148	IRR	7(3/1/3)
28	Chen 2018 [53]	RA	China	RC	NHIRD	2006–2011	18–45	74.05%	ICD-9	ICD-9	10568	IRR	8(3/2/3)
29	Curtis 2018 [33]	RA	USA	RC	MPCD	2006–2010	40–85	80.00%	ICD-9	ICD-9	494	IRR	8(3/2/3)
30	Lee 2018 [54]	AS	South Korea	RC	KNHIS	2010–2014	>20	27.46%	ICD-10	ICD-10	12988	IRR	8(3/2/3)
31	Tsai 2018 [55]	Gout	China	RC	NHI	2000–2005	NA	NA	NA	NA	646983	HR	6(3/1/2)
32	Kasai 2019 [56]	RA	Janpan	RC	JMDC	2005–2014	≥18	75.60%	ICD-10	ICD-10	6712	IRR	7(3/2/2)

ref.no, reference number; ACR, American College of Rheumatology; AMORIS, Apolipoprotein Mortality RISk; ARA, American Rheumatism Association; ARAMIS, Arthritis, Rheumatism, and Aging Medical Information System; AS, ankylosing spondylitis; BC, British Columbia; BCLHD, British Columbia Longitudinal Health Database; DCVS, Danish civil registration system; ERAS, Early RA Study; FHS, Framingham Heart Study; GPRD, General Practice Research Database; HER, electronic health record; JMDC, Japan Medical Data Center; KNHIS, Korean NationalHealthInsurance Service; LHID, Longitudinal Health Insurance Database; MPCD, multi-payer claims database; NHI, National Health Insurance; NHIRD, National Health Insurance Research Database; NHS, National Health Service; NOS, Newcastle-Ottawa Scale; NPR&SPR, The National Patient Register and the Swedish Population Register; NSDR, National Swedish data registers; OA, osteoarthritis; ORLS, Oxford Record Linkage Study; PC, prospective cohort study; PsA, psoriatic arthritis; RA, Rheumatoid arthritis; RAMQ, Re´gie de l’Assurance Maladie du Que´bec database; RC, retrospective cohort study; SCHS, Singapore Chinese Health Study; SHDR, Swedish Hospital Discharge Register; SNPR, Swedish National Patient Register; THIN, The Health Improvement Network; TLHID, Taiwan’s Longitudinal Health Insurance Database.

Some articles contained several sub-cohorts; if the researchers only provided the RR and 95% CI for each sub-cohort but not the RR and 95% CI for the total cohort, we treated each sub-cohort as an independent cohort study in the meta-analysis. In this way, the 32 articles contained a total of 52 independent cohort studies. Among them, 29 studies were on RA, 4 on OA, 10 on AS, 4 on PsA, 5 on gout, and 31 studies adjusting for at least one traditional RF. The covariates adjusted for each study are shown in S1 Table.

As seen in Table 1, the quality of the 31 included articles was high, with only one article being judged as moderate quality.

### Stroke risk for all arthritis types

**Fig 2:**
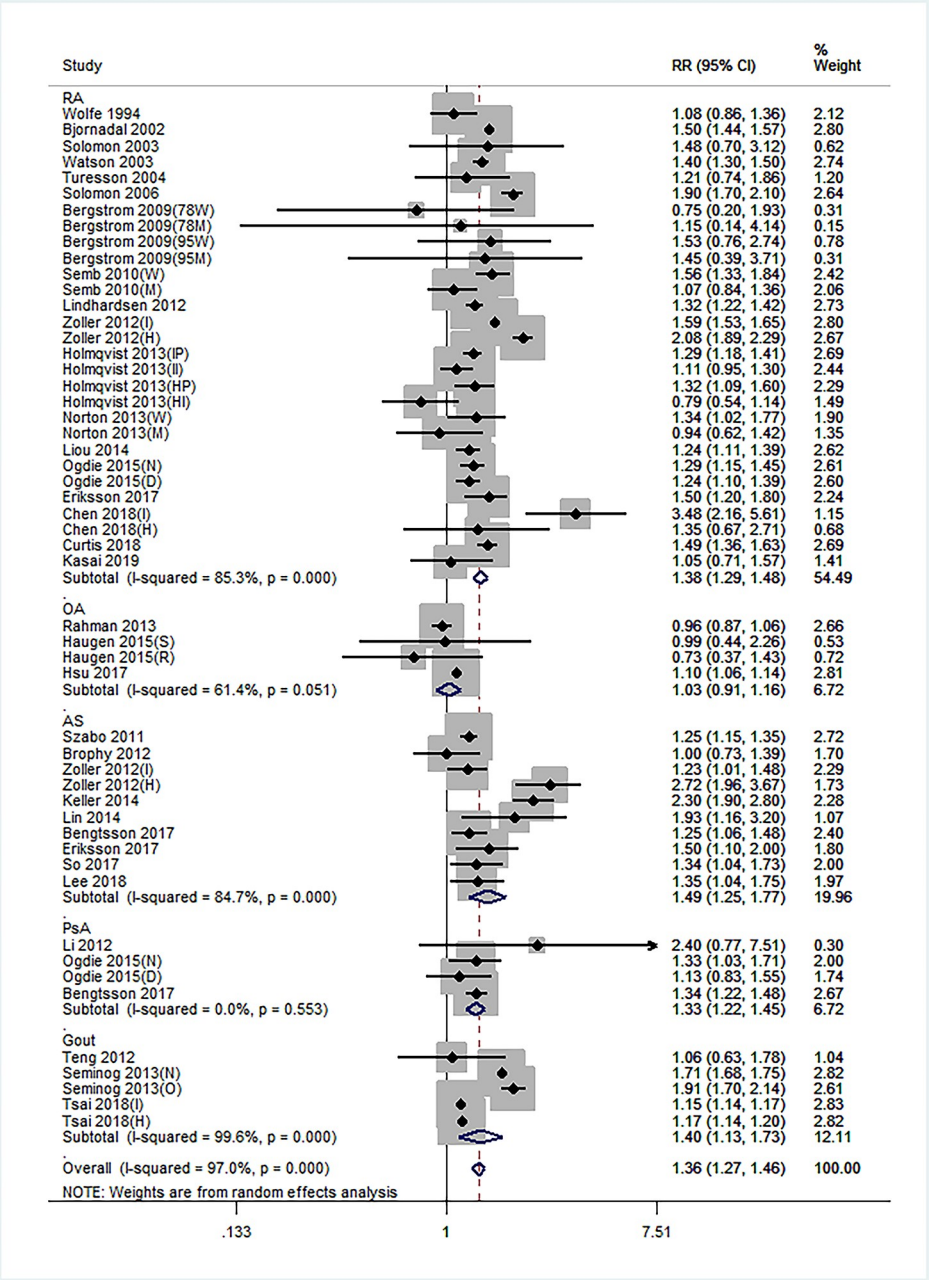
Forest plot showing the stroke risk in arthritis in studies adjusted for age and sex.

Of the 52 independent cohort studies, 31 (59.62%) reported an increased risk of stroke in arthritis. The combined results showed that the stroke risk in arthritis was significantly increased in studies adjusting for age and sex only (RR = 1.36, 95% CI: 1.27–1.46) and for at least one traditional RF (RR = 1.40, 95% CI: 1.28–1.54). A random-effects model was used, owing to the high heterogeneity between studies (I^2^ = 97%) (Fig 2).

### Stroke risk for each arthritis type

**Fig 3:**
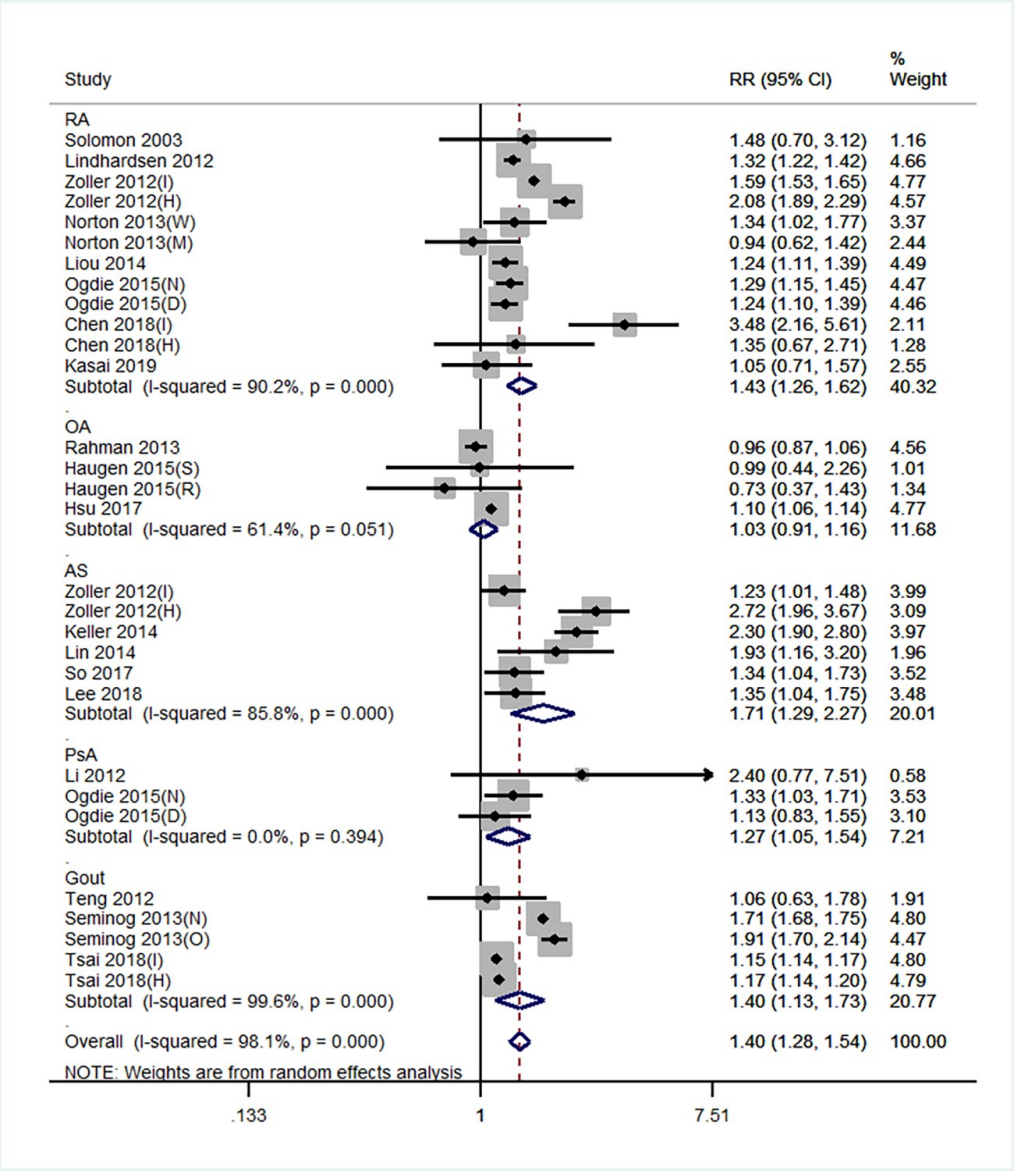
Forest plot showing the stroke risk in arthritis in studies adjusted for age and sex and at least one traditional risk factor (hypertension, diabetes, smoking, alcoholism, obesity, physical inactivity, and hyperlipidemia).

In subgroup analysis according to arthritis type, the results based on studies adjusting for age and sex revealed that stroke risk was significantly increased in RA (RR = 1.38, 95% CI: 1.29–1.48), AS (RR = 1.49, 95% CI: 1.25–1.77), PsA (RR = 1.33, 95% CI: 1.22–1.45), and gout (RR = 1.40, 95% CI: 1.13–1.73); stroke risk was not significantly increased in OA (RR = 1.03, 95% CI: 0.91–1.16). In studies adjusting for at least one traditional RF, the results showed no obvious change compared with those adjusting for age and sex (Fig 3).

### Risk of stroke subtype for all arthritis types

In the 52 studies adjusting for age and sex, 13 separately analyzed the risk of IS, and 6 analyzed the risk of HS; the results suggested that the risk of IS and HS was significantly increased in arthritis (IS: RR = 1.53, 95% CI: 1.32–1.78; HS: RR = 1.45, 95% CI: 1.15–1.84). Of the studies adjusting for at least one traditional RF, the risk of IS and HS was also significantly increased (IS: RR = 1.59, 95% CI: 1.32–1.92; HS: RR = 1.63, 95% CI: 1.23–2.15) (Table 2).

**Table 2:** Subgroup analyses of population-based studies estimating stroke risk in the major types of arthritis.

Comparison	Studies adjusting for age and sex only			Studies adjusting for age, sex and at least one traditional RF^#^		
	Study number	Random-effects RR (95% CI)	*P* value	Study number	Random-effects RR (95% CI)	*P* value
**Stroke Type**						
IS	14	1.53(1.32, 1.78)	<0.001	10	1.59(1.32, 1.92)	<0.001
HS	7	1.45(1.15, 1.84)	<0.001	5	1.63(1.23, 2.15)	<0.001
**Sex**						
Female	20	1.47(1.31, 1.66)	<0.001	11	1.68(1.43, 1.97)	<0.001
Male	20	1.44(1.28, 1.61)	<0.001	11	1.53(1.33, 1.77)	<0.001
**Age**						
<45	4	1.46(1.17, 1.82)	0.001	2	1.60(0.70, 3.62)	0.262*
45–64	11	1.43(1.18, 1.72)	<0.001	5	1.57(1.19, 2.07)	0.001
≥65	10	1.17(1.08, 1.26)	<0.001	4	1.12(1.04, 1.21)	0.004
**Cohort Type**						
PC	16	1.26(1.17, 1.35)	<0.001	7	1.30(1.08, 1.57)	0.005
RC	36	1.42(1.31, 1.53)	<0.001	23	1.42(1.28, 1.57)	<0.001
**Region**						
Asia	11	1.26(1.18, 1.35)	<0.001	11	1.26(1.18, 1.35)	<0.001
Europe	31	1.39(1.31, 1.48)	<0.001	13	1.50(1.38, 1.64)	<0.001
North America	10	1.28(1.06, 1.55)	0.011	6	1.11(0.88, 1.41)	0.386*
**Publication Date**						
≤2009	10	1.46(1.29, 1.64)	<0.001	1	1.48(0.70, 3.12)	0.304*
≥2010	42	1.35(1.26, 1.46)	<0.001	29	1.40(1.28, 1.54)	<0.001
**Effect Estimate**						
IRR	29	1.39(1.26, 1.54)	<0.001	19	1.39(1.20, 1.60)	<0.001
SIR	7	1.53(1.28, 1.81)	<0.001	6	1.59(1.31, 1.93)	<0.001
HR	9	1.18(1.14, 1.23)	<0.001	5	1.16(1.13, 1.20)	<0.001
SMR	7	1.31(1.09, 1.57)	0.004	NA	NA	NA

RR, relative risk; PC, prospective cohort study; RC, retrospective cohort study; SMR, standardized mortality rate; SIR, standardized incidence ratio; IRR, incidence rate ratio; HR, hazard ratio; RF, risk factor; NA, not applicable.

* No statistical significance.

^#^traditional RF: hyperlipidemia, diabetes, high blood pressure, smoking, obesity and physical activity.

### Age and sex subgroup analyses

In subgroup analysis based on sex, the risk was similar (women: RR = 1.47, 95% CI: 1.31–1.66; men: RR = 1.44, 95% CI: 1.28–1.61). Subgroup analysis stratified by age revealed that stroke risk was highest with younger age (<45 years) (RR = 1.46, 95% CI: 1.17–1.82) and relatively lower with older age (≥65 years) (RR = 1.17, 95% CI: 1.08–1.26) (Table 2).

### Region and cohort type subgroup analyses

In the region-based subgroup analysis, the stroke risk was similar in Asia and Europe but slightly higher in North America (Asia: RR = 1.26,95%CI:1.18–1.35; Europe: RR = 1.39,95%CI:1.31–1.48; North America: RR = 1.28,95%CI:1.06–1.55). Subgroup analysis by cohort study type showed that RC studies was slightly higher than PC studies (RC: RR = 1.42,95%CI:1.31–1.53; PC: RR = 1.26,95%CI:1.17–1.35) (Table 2).

### Heterogeneity testing and sensitivity analysis

The combined analysis of 52 studies showed high heterogeneity (I^2^ = 97%, *p* < 0.001). To explore the source of heterogeneity, we first performed subgroup analyses by arthritis type, stroke subtype, sex, age, effect estimate type, region, cohort study type, publication date. However, the results showed that they all could not explain the source of heterogeneity (Table 2). We further performed sensitivity analyses to investigate the source of significant heterogeneity and to test the impact of each single study on the final results. We found that no single study qualitatively changed the pooled effects, indicating the reliability and stability of our results. We also found that no single study had a significant effect on heterogeneity; with the I^2^ statistic reduced to < 50%, 14 studies had to be excluded.

### Publication bias

**Fig 4:**
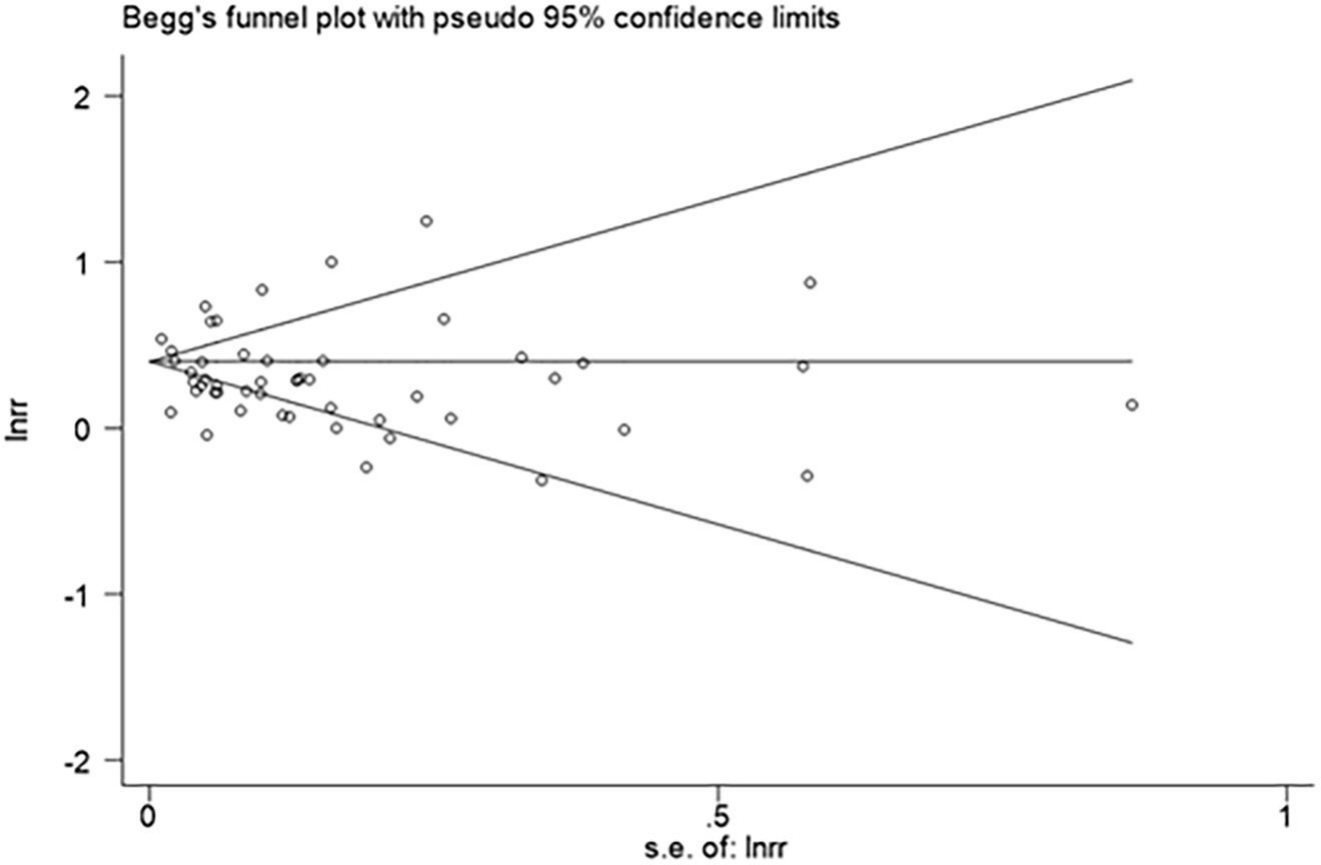
Funnel plot for stroke risk in arthritis.

The Egger test (*p* = 0.435) and Begg test (*p* = 0.313) were not statistically significant, and the funnel plot was roughly symmetrical, indicating that no significant publication bias was present (Fig 4).

## Discussion

In the past few decades, numerous studies on risk factors for stroke have focused on traditional RF such as hypertension, diabetes, obesity, smoking, alcoholism, and physical inactivity. There has been a growing number of studies on new RF for stroke in recent years. However, the sample size in most studies was small and the conclusions were inconsistent. Therefore, some researchers merged multiple studies in quantitative meta-analyses and found a series of risk factors involved in stroke, including migraine, anemia, inflammatory bowel disease, sleep insufficiency, insufficient intake of fruits and vegetables, and inflammatory diseases [57–61]. Wiseman et al. [17] conducted a meta-analysis of studies on stroke risk in rheumatic diseases and found that RA, AS, gout, and systemic lupus erythematosus increased stroke risk to varying degree. Since these studies, there have been no meta-analyses of stroke risk in any arthritis type, which may lead to underestimation of the actual stroke risk in clinical practice.

To date, our review is the most comprehensive systematic review and meta-analysis of published cohort studies to evaluate the stroke risk in arthritis. We compared the stroke risk in multiple types of arthritis, not only adjusting for age and sex but also adjusting for traditional RF, which may be confounding factors for arthritis. Our review suggested that individuals with arthritis had a 36% higher risk of developing stroke than the general population. This relationship was also found in stroke subgroup analysis, with a 53% higher risk of IS and a 45% higher risk of HS. Compared with middle-aged and older patients, younger patients had the highest stroke risk. Interestingly, a few years ago, Fransen et al. [62] performed a similar meta-analysis in which they found a higher CVD risk among younger RA patients. As young people ordinarily have fewer traditional RF, we speculate that arthritis is an independent risk factor that does not depend on traditional RF. Besides, In 2017, Schieir et al. [63] conducted a meta-analysis on the risk of myocardial infarction (MI) in arthritis. They found that the MI risk in all types of arthritis was attenuated in studies adjusting for traditional RF, in comparison with unadjusting studies, further suggesting that traditional RF could partially explain the MI risk in arthritis. Unlike their conclusion, our review showed that traditional RF could not explain the stroke risk in arthritis because this risk adjusting for traditional RF was slightly higher than the stroke risk in arthritis without adjustment for traditional RF. Taken together, we have good reasons to consider arthritis as an independent risk factor for stroke.

Owing to the rising incidence rate of arthritis, our review is of great clinical importance [64]. First, we assessed the implications of the review from the perspective of patients. Stroke risk in RA has been widely studied and is generally widely recognized [65], whereas stroke risk in other types of arthritis has received much less attention and may not be fully recognized, especially in OA and PsA. We found an increased risk of stroke in the most common types of arthritis; therefore, stroke can be considered a common complication of arthritis and should be taken more seriously. Second, OA is generally considered non-systemic inflammatory arthritis; the other four types of arthritis (RA, AS, PsA, gout) are considered systemic inflammatory arthritis. Our review showed that OA has no additional risk of stroke, suggesting that systemic inflammation may be the direct cause of increased risk of stroke. From the clinician’s perspective, this information is essential because clinicians can prioritize the control of inflammation among traditional RF, which may help to reduce the stroke risk. Lastly, the association between arthritis and stroke is important from a public health perspective. It is necessary to consider screening arthritis status and RF for cardio-cerebrovascular diseases in the general population for early intervention to reduce future stroke events.

The strengths of our review include the following: (i) because of cohort studies’ powerful ability to test the RF hypothesis, we only included cohort studies in our meta-analysis, rendering our conclusions relatively more reliable. (ii) compared with a study by Wiseman et al., we conducted a more comprehensive search and had a much larger sample size and broader study area, compensating for the lack of information on Asian patients with arthritis in that previous analysis. (iii) for the first time, we compared the stroke risk for multiple types of arthritis in studies adjusting for age and sex, which are the most common confounding factors, then studies adjusting for traditional RF, which helps in determining whether arthritis is a RF independent of traditional RF.

This review includes several limitations: (i) there was a high degree of heterogeneity between studies. Although we conducted subgroup analyses and sensitivity analyses, we were unable to identify potential sources of heterogeneity. We assumed that the statistical heterogeneity was primarily attributable to the degree of variability in effect size across different studies; therefore, we used a random-effects model to explain variability across studies. (ii) although we have excluded explicitly duplicated studies, there may be some overlap among the populations of more than one study from the same cohort. (iii) 69% of the included studies were retrospective, this may lead to biases inherent in these types of studies.

More than 60% of the included studies did not explore stroke subtypes, more than 50% did not carry out age stratification, and more than 40% did not adjust for any traditional RF. Therefore, additional large-sample cohort studies are needed to explore the effects of different types of arthritis on stroke and other vascular end-point events; such studies should be stratified by stroke subtype, age, and sex and should adjust for traditional RF. In addition, further clinical studies are also needed to identify improved treatments for different types of arthritis.

## Conclusions

Although observational studies cannot prove causality, the findings of this meta-analysis of large, high-quality population-based cohort studies strongly support that multiple types of arthritis increase stroke risk. These findings provide additional reliable evidence for arthritis as an independent RF for stroke, which requires greater attention among patients with arthritis. However, considering the potential bias and confounding in the included studies, caution should be exercised in the interpretation of the results.

## Supporting information

S1 Checklist PRISMA 2009 checklist used in this meta-analysis. (DOCX)

S1 Appendix Sample MEDLINE search strategy. (DOCX)

S1 Table The covariates for adjustment in each study. (DOCX)
