# Supplementary material for: Stroke risk in arthritis: A systematic review and meta-analysis of cohort studies
Source: PLoS One. 2021 Mar 16;16(3):e0248564. doi: 10.1371/journal.pone.0248564 (PMC7963101; doi:10.1371/journal.pone.0248564)
Supplement: S1 Appendix — (DOCX) [file pone.0248564.s002.docx]

**S1 Appendix**

**Sample MEDLINE search strategy**

((((("Stroke"[Mesh]) OR ((((((((((((((((((((((((((((((((((((((((((((((((((((((((((((((Accident, Cerebrovascular[Title/Abstract]) OR Acute Cerebrovascular Accident[Title/Abstract]) OR Acute Cerebrovascular Accidents[Title/Abstract]) OR Acute Cerebrovascular Lesion[Title/Abstract]) OR Acute Focal Cerebral Vasculopathy[Title/Abstract]) OR Acute Stroke[Title/Abstract]) OR Acute Strokes[Title/Abstract]) OR Acute Stroke[Title/Abstract]) OR Apoplectic Stroke[Title/Abstract]) OR Apoplexia[Title/Abstract]) OR Apoplexy[Title/Abstract]) OR Apoplexy, Cerebrovascular[Title/Abstract]) OR Blood Flow Disturbance, Brain[Title/Abstract]) OR Brain Accident[Title/Abstract]) OR Brain Attack[Title/Abstract]) OR Brain Blood Flow Disturbance[Title/Abstract]) OR Brain Insult[Title/Abstract]) OR Brain Insultus[Title/Abstract]) OR Brain Ischaemic Attack[Title/Abstract]) OR Brain Ischemic Attack[Title/Abstract]) OR Brain Vascular Accident[Title/Abstract]) OR Brain Vascular Accidents[Title/Abstract]) OR Cerebral Apoplexia[Title/Abstract]) OR Cerebral Insult[Title/Abstract]) OR Cerebral Stroke[Title/Abstract]) OR Cerebral Strokes[Title/Abstract]) OR Cerebral Vascular Accident[Title/Abstract]) OR Cerebral Vascular Insufficiency[Title/Abstract]) OR Cerebral Stroke[Title/Abstract]) OR Cerebro Vascular Accident[Title/Abstract]) OR Cerebrovascular Accident[Title/Abstract]) OR Cerebrovascular Accident, Acute[Title/Abstract]) OR Cerebrovascular Accidents[Title/Abstract]) OR Cerebrovascular Accidents, Acute[Title/Abstract]) OR Cerebrovascular Apoplexy[Title/Abstract]) OR Cerebrovascular Arrest[Title/Abstract]) OR Cerebrovascular Failure[Title/Abstract]) OR Cerebrovascular Injury[Title/Abstract]) OR Cerebrovascular Insufficiency[Title/Abstract]) OR Cerebrovascular Insult[Title/Abstract]) OR Cerebrovascular Strokes[Title/Abstract]) OR Cerebrovascular Stroke[Title/Abstract]) OR Cerebrum Vascular Accident[Title/Abstract]) OR Cryptogenic Stroke[Title/Abstract]) OR CVA[Title/Abstract]) OR CVA (Cerebrovascular Accident)[Title/Abstract]) OR CVAs[Title/Abstract]) OR CVAs (Cerebrovascular Accident)[Title/Abstract]) OR Ischaemic Cerebral Attack[Title/Abstract]) OR Ischaemic Seizure[Title/Abstract]) OR Ischemic Cerebral Attack[Title/Abstract]) OR Ischemic Seizure[Title/Abstract]) OR Stroke, Acute[Title/Abstract]) OR Stroke, Cerebral[Title/Abstract]) OR Stroke, Cerebrovascular[Title/Abstract]) OR Strokes[Title/Abstract]) OR Strokes, Acute[Title/Abstract]) OR Strokes, Cerebral[Title/Abstract]) OR Strokes, Cerebrovascular[Title/Abstract]) OR Vascular Accident, Brain[Title/Abstract]) OR Vascular Accidents, Brain[Title/Abstract]))))) **AND** (((((((("Arthritis"[Mesh]) OR ((((((((((Arthritides[Title/Abstract]) OR Arthrochondritis[Title/Abstract]) OR Arthrosynovitis[Title/Abstract]) OR Chronic Senescent Arthritis[Title/Abstract]) OR Joint Inflammation[Title/Abstract]) OR Oligoarthritis[Title/Abstract]) OR Polyarthritides[Title/Abstract]) OR Polyarthritis[Title/Abstract]) OR Sero Negative Arthritis[Title/Abstract]) OR Undifferentiated Arthritis[Title/Abstract]))) OR (("Arthritis, Rheumatoid"[Mesh]) OR (((((((((((((((((((Arthritis Deformans[Title/Abstract]) OR Arthrosis Deformans[Title/Abstract]) OR Beauvais Disease[Title/Abstract]) OR Chronic Polyarthritis[Title/Abstract]) OR Chronic Progressive Poly Arthritis[Title/Abstract]) OR Chronic Progressive Polyarthritis[Title/Abstract]) OR Chronic Rheumatoid Arthritis[Title/Abstract]) OR Disease, Beauvais[Title/Abstract]) OR Infantile Rheumatoid Arthritis[Title/Abstract]) OR Inflammatory Arthritis[Title/Abstract]) OR Polyarthritis, Primary Chroni[Title/Abstract]) OR Primary Chronic Polyarthritis[Title/Abstract]) OR Progressive Polyarthritis, Chronic[Title/Abstract]) OR Rheumarthritis[Title/Abstract]) OR Rheumatic Arthritis[Title/Abstract]) OR Rheumatic Polyarthritis[Title/Abstract]) OR Rheumatism, Chronic Articular[Title/Abstract]) OR Rheumatoid Arthritis[Title/Abstract]))) OR (("Arthritis, Psoriatic"[Mesh]) OR (((((((((((((((((((((Alibert Bazin Disease[Title/Abstract]) OR Arthritic Psoriasis[Title/Abstract]) OR Arthritis Psoriatica[Title/Abstract]) OR Arthritis, Psoriasis[Title/Abstract]) OR Arthritis, Psoriatic[Title/Abstract]) OR Arthropathies, Psoriatic[Title/Abstract]) OR Arthropathy, Psoriatic[Title/Abstract]) OR Disease, Alibert Bazin[Title/Abstract]) OR Polyarthritis, Psoriatic[Title/Abstract]) OR Psoriasis Arthropathica[Title/Abstract]) OR Psoriasis Pustulosa Arthropathica[Title/Abstract]) OR Psoriasis, Arthritic[Title/Abstract]) OR Psoriasis, Arthritis[Title/Abstract]) OR Psoriatic Arthritis[Title/Abstract]) OR Psoriatic Arthropathies[Title/Abstract]) OR Psoriatic Arthropathy[Title/Abstract]) OR Psoriatic Polyarthritis[Title/Abstract]) OR Psoriatic Rheumatism[Title/Abstract]) OR Psoriatic Rheumatoid Arthritis[Title/Abstract]) OR Rheumatoid Arthritis, Psoriatic[Title/Abstract]))) OR (("Spondylitis, Ankylosing"[Mesh]) OR (((((((((((((((((((((((((((((((((((Ankylating Spondylitis[Title/Abstract]) OR Ankylopoietic Spondylarthritis[Title/Abstract]) OR Ankylopoietic Spondylitis[Title/Abstract]) OR Ankylosing Spine[Title/Abstract]) OR Ankylosing Spondylarthritides[Title/Abstract]) OR Ankylosing Spondylarthritis[Title/Abstract]) OR Ankylosing Spondylarthrosis[Title/Abstract]) OR Ankylosing Spondylitis[Title/Abstract]) OR Ankylosing Spondyloarthritides[Title/Abstract]) OR Ankylosing Spondyloarthritis[Title/Abstract]) OR Ankylosis Spondylitis[Title/Abstract]) OR Ankylotic Spondylitis[Title/Abstract]) OR Bechterew Disease[Title/Abstract]) OR Bechterews Disease[Title/Abstract]) OR Bechterew's Disease[Title/Abstract]) OR Bekhterev Disease[Title/Abstract]) OR Marie Struempell Disease[Title/Abstract]) OR Marie-Struempell Disease[Title/Abstract]) OR Morbus Bechterew[Title/Abstract]) OR Rheumatoid Spondylitis[Title/Abstract]) OR Spinal Ankylosis[Title/Abstract]) OR Spine Ankylosis[Title/Abstract]) OR Spondylarthritides, Ankylosing[Title/Abstract]) OR Spondylarthritis Ankylopoietica[Title/Abstract]) OR Spondylarthritis Ankylosans[Title/Abstract]) OR Spondylarthritis, Ankylosing[Title/Abstract]) OR Spondylarthrosis Ankylopoietica[Title/Abstract]) OR Spondylitis Ankylopoetica[Title/Abstract]) OR Spondylitis, Rheumatoid[Title/Abstract]) OR Spondyloarthritides, Ankylosing[Title/Abstract]) OR Spondyloarthritis Ankylopoietica[Title/Abstract]) OR Spondyloarthritis, Ankylosing[Title/Abstract]) OR Vertebral Ankylosis[Title/Abstract]))) OR (("Gout"[Mesh]) OR ((((((((((Arthragra[Title/Abstract]) OR Arthritis Urica[Title/Abstract]) OR Arthritis, Gouty[Title/Abstract]) OR Cheiragra[Title/Abstract]) OR Chiragra[Title/Abstract]) OR Gouts[Title/Abstract]) OR Gouty Arthritis[Title/Abstract]) OR Gouty Attack[Title/Abstract]) OR Urate Inflammation[Title/Abstract]) OR Uric Arthritis[Title/Abstract]))) OR (("Osteoarthritis"[Mesh]) OR ((((((((((((((((Arthritides, Degenerative[Title/Abstract]) OR Arthritis, Degenerative[Title/Abstract]) OR Arthritis, Noninflammatory[Title/Abstract]) OR Arthroses[Title/Abstract]) OR Arthrosis[Title/Abstract]) OR Degenerative Arthritides[Title/Abstract]) OR Degenerative Arthritis[Title/Abstract]) OR Degenerative Joint Disease[Title/Abstract]) OR Osteoarthritides[Title/Abstract]) OR Osteo-Arthritis[Title/Abstract]) OR Osteoarthroses[Title/Abstract]) OR Osteoarthrosis[Title/Abstract]) OR Osteo-Arthrosis[Title/Abstract]) OR Osteoarthrosis Deformans[Title/Abstract]) OR Primary Osteoarthritis[Title/Abstract]) OR Rheumatoid Arthrosis[Title/Abstract]))) OR (("Chondrocalcinosis"[Mesh]) OR ((((((((((((Chondrocalcinoses[Title/Abstract]) OR Pseudogout[Title/Abstract]) OR Calcium Pyrophosphate Deposition Disease[Title/Abstract]) OR Calcium Pyrophosphate Dihydrate Deposition[Title/Abstract]) OR articular chondrocalcinosis[Title/Abstract]) OR chondrocalcinosis articularis[Title/Abstract]) OR chondrocalcinosis, articular[Title/Abstract]) OR chondrocalcinosis, joint[Title/Abstract]) OR joint chondrocalcinosis[Title/Abstract]) OR pseudo podagra[Title/Abstract]) OR pseudogout syndrome[Title/Abstract]) OR pseudopodagra[Title/Abstract])))) **AND** (("Cohort Studies"[Mesh]) OR (((((((((((((((((((((((((((((((((((((((((((((((((((((((((((Analyses, Cohort[Title/Abstract]) OR Analysis, Cohort[Title/Abstract]) OR Closed Cohort Study[Title/Abstract]) OR Closed Cohort Studies[Title/Abstract]) OR Cohort Analyses[Title/Abstract]) OR Cohort Analysis[Title/Abstract]) OR Cohort Fertility[Title/Abstract]) OR Cohort Life Cycle[Title/Abstract]) OR Cohort Studies, Closed[Title/Abstract]) OR Cohort Studies, Historical[Title/Abstract]) OR Cohort Studies, Prospective[Title/Abstract]) OR Cohort Studies, Retrospective[Title/Abstract]) OR Cohort Study[Title/Abstract]) OR Cohort Study, Closed[Title/Abstract]) OR Cohort Study, Historical[Title/Abstract]) OR Cohort Study, Prospective[Title/Abstract]) OR Cohort Study, Retrospective[Title/Abstract]) OR Concurrent Studies[Title/Abstract]) OR Concurrent Study[Title/Abstract]) OR Fertility, Cohort[Title/Abstract]) OR Follow-Up Studies[Title/Abstract]) OR Follow-Up Study[Title/Abstract]) OR Historical Cohort Study[Title/Abstract]) OR Historical Cohort Studies[Title/Abstract]) OR Incidence Studies[Title/Abstract]) OR Incidence Study[Title/Abstract]) OR Longitudinal Studies[Title/Abstract]) OR Longitudinal Study[Title/Abstract]) OR Mortality Studies[Title/Abstract]) OR Mortality Study[Title/Abstract]) OR Prospective Cohort Study[Title/Abstract]) OR Prospective Studies[Title/Abstract]) OR Prospective Study[Title/Abstract]) OR Prospective Cohort Studies[Title/Abstract]) OR Retrospective Cohort Study[Title/Abstract]) OR Retrospective Cohort Studies[Title/Abstract]) OR Studies, Closed Cohort[Title/Abstract]) OR Studies, Cohort[Title/Abstract]) OR Studies, Concurrent[Title/Abstract]) OR Studies, Follow-Up[Title/Abstract]) OR Studies, Historical Cohort[Title/Abstract]) OR Studies, Incidence[Title/Abstract]) OR Studies, Longitudinal[Title/Abstract]) OR Studies, Mortality[Title/Abstract]) OR Studies, Prospective[Title/Abstract]) OR Studies, Prospective Cohort[Title/Abstract]) OR Studies, Retrospective Cohort[Title/Abstract]) OR Study, Closed Cohort[Title/Abstract]) OR Study, Cohort[Title/Abstract]) OR Study, Concurrent[Title/Abstract]) OR Study, Follow-Up[Title/Abstract]) OR Study, Historical Cohort[Title/Abstract]) OR Study, Incidence[Title/Abstract]) OR Study, Longitudinal[Title/Abstract]) OR Study, Mortality[Title/Abstract]) OR Study, Prospective[Title/Abstract]) OR Study, Prospective Cohort[Title/Abstract]) OR Study, Retrospective Cohort[Title/Abstract]))
