# Supplementary material for: Stroke risk in arthritis: A systematic review and meta-analysis of cohort studies
Source: PLoS One. 2021 Mar 16;16(3):e0248564. doi: 10.1371/journal.pone.0248564 (PMC7963101; doi:10.1371/journal.pone.0248564)
Supplement: S1 Table — (DOCX) [file pone.0248564.s003.docx]

**S1 Table. The additional covariates for adjustment in each study**

| **Study** | **Additional covariates** |
| --- | --- |
| Wolfe 1994 | age, sex |
| Bjornadal 2002 | age, sex |
| Solomon 2003 | age, sex, hypertension,diabetes, high cholesterol level, parental history of myocardial,infarction before age 60 years, BMI, cigarette use, physical activity, alcohol use, aspirin use, vitamin E supplement intake, antiinflammatory drug use, folate intake, omega-3 fatty acid intake,replacement therapy use, oral glucocorticoid use, nonsteroidalmenopausal status, hormone |
| Watson 2003 | age, sex |
| Turesson 2004 | age, sex |
| Solomon 2006 | age, sex |
| Bergstrom 2009 | age, sex |
| Semb 2010 | age, sex |
| Szabo 2011 | age, sex |
| Brophy 2012 | age, sex |
| Li 2012 | age, sex, BMI, weight, overweight, obesity, smoking, alcohol, race, family history, hypertension, hypercholesterolaemia, current aspirin use, multivitamin use, postmenopausal hormone use, oral contraceptives use |
| Lindhardsen 2012 | age, sex, calendar year, socioeconomic status, hypertension, heart failure, ischaemic heart disease, hyperthyroid disease, COPD, heart surgery, diabetes, cardiovascular drugs |
| Teng 2012 | age, sex, years between baseline and follow up, BMI,gender, dialect group, education,alcohol consumption,physical activity, cigarette smoking, dietary saturated fat density, dietary cholesterol density, diabeteshypertension, |
| Zoller 2012 | age, sex, period, socioeconomic status, region of residence, hospitalization of chronic lower respiratory diseases, obesity, alcoholism, hypertension, diabetes, atrial fibrillation, heart failure, renal disease, sepsis, and CHD |
| Holmqvist 2013 | age, sex |
| Norton 2013 | age, sex, social class, smoking, erosions, functional grade, RF, ANA, BMI, ESR, visual analogue scale pain, HAQ, DAS, hypertension, and time to first DMARD and steroid use within the first year |
| Rahman 2013 | age, sex, family history, high cholesterol, high blood pressure, diabetes mellitus, BMI, smoking, and diet |
| Seminog 2013 | age, sex, time period in single calendar years and for region of residence and deprivation score associated with patient’s area of residence, in quintiles. |
| Keller 2014 | age, sex, chronic lower respiratory diseases, type 2 diabetes mellitus, hypertension, hyperlipidemia, heart failure, renal disease, sepsis, coronary heart disease, atrial fibrillation and log-income |
| Lin 2014 | age, sex, hypertension, CHD |
| Liou 2014 | age, sex, urbanization level, diabetes mellitus, hypertension, hyperlipidemia, coronary heart diseases, congestive heart failure, hypercoagulability, renal disease, atrial fibrillation, valvular heart disease, Aspirin use, Hydroxychloroquine use and NSAIDs use |
| Haugen 2015 | age, sex, cohort, BMI, total cholesterol:HDL ratio, current lipid lowering treatment, increased blood pressure, current antihypertensive treatment, elevated fasting or non-fasting blood glucose, current antidiabetic treatment (oral or insulin), current use of NSAIDs, daily use of aspirin, current/previous smoking, alcohol use |
| Ogdie 2015 | age, sex, hypertension, diabetes, hyperlipidaemia, smoking status (never, past, current) and start year in the cohort |
| Bengtsson 2017 | age, sex |
| Eriksson 2017 | age, sex |
| Hsu 2017 | age, sex, NSAIDs, and comorbidiYes of hypertension, diabetes, hyperlipidemia, CAD, obesity, congesYve heart failure, and alcoholism |
| So 2017 | age, sex, obesity, angina, COPD, hospitalizations in year before index date, Charlson’s comorbidity index, oral glucocorticoids, cardiovascular drugs, anti-diabetic medication, HRT, contraceptives, fibrates, statins, NSAIDs, and Cox-2 inhibitors |
| Chen 2018 | age, sex, hypertension, diabetes mellitus, hyperlipidemia, chronic kidney disease, cancer, peripheral vascular disease, mild liver disease, chronic obstructive pulmonary disease, obesity, anticoagulants, antiplatelets, DMARD, NSAIDs, glucocorticoids, and biologics |
| Curtis 2018 | age, sex |
| Lee 2018 | age, sex, income, diabetes, hypertension, and dyslipidemia |
| Tsai 2018 | age, sex, Charlson comorbidity index and co-medication |
| Kasai 2019 | age, sex, hypertension, diabetes mellitus, dyslipidaemia,chronic kidney disease, and stroke at baselin |

ANA, antinuclear antibody; BMI, body mass index; CAD, coronary artery disease; CHD, coronary heart disease; COPD, chronic obstructive pulmonary disease; DAS, disease activity score; DMARD, disease-modifying anti-rheumatic drugs; ESR, erythrocyte sedimentation rate; HAQ, health assessment questionnaire; NSAIDs, nonsteroidal antiinflammatory drugs; RF, rheumatoid factor.
